# ASO Author Reflections: Surgical Strategy and Recurrence Patterns in HCC: Anatomical Resection Reduces Risk of Non-transplantable Recurrence

**DOI:** 10.1245/s10434-025-17454-y

**Published:** 2025-05-10

**Authors:** Jun Kawashima, Miho Akabane, Timothy M. Pawlik

**Affiliations:** 1 Department of Surgery, The Ohio State University Wexner Medical Center and James Comprehensive Cancer Center, Columbus, OH USA; 2 Department of Gastroenterological Surgery, Yokohama City University School of Medicine, Yokohama, Japan

## Past

Hepatic resection has long been the cornerstone of curative-intent treatment for patients with resectable hepatocellular carcinoma (HCC).^1^ Nevertheless, recurrence following resection occurs in up to 50–70% of patients, underscoring the importance of effective strategies for managing recurrent disease.^2^ Among the various patterns of recurrence, non-transplantable recurrence (NTR)—defined as recurrence beyond the Milan criteria—represents a particularly challenging scenario due to the limited availability of curative options such as salvage liver transplantation or repeat hepatectomy.^3^ While the debate between anatomical resection (AR) and non-anatomical resection (NAR) continues, few studies have specifically examined the impact of surgical approach on the risk of developing NTR.^4^ Therefore, the aim of the current study was to clarify the influence of initial surgical strategy—AR versus NAR—on the risk of NTR among patients with HCC.^5^

## Present

We analyzed 1038 patients who underwent curative-intent liver resection for HCC within the Milan criteria across multiple international institutions in both Western and Eastern countries. Among these patients, 747 (72.0%) underwent AR, while 291 (28.0%) underwent NAR. After adjustment using inverse probability of treatment weighting, patients who underwent AR demonstrated a lower risk of NTR compared with individuals who had a NAR (3-year NTR 9.8% versus 14.4%, HR 0.62, 95% CI 0.40–0.96). Importantly, subgroup analysis demonstrated that the benefit of AR was most pronounced among patients with a medium tumor burden score (TBS), whereas the advantage was less evident among patients with low TBS. These results suggested that the choice of surgical approach may have differential implications on the basis of tumor morphology and highlight the potential oncologic benefit of AR, particularly among patients with a greater tumor burden.

## Future

Given the poor prognosis and limited treatment options associated with NTR, future studies should continue to refine our understanding of how surgical strategy affects the biology of recurrence. Integration of tumor biology, morphologic metrics such as TBS, and emerging biomarkers may help identify subgroups most likely to benefit from AR. Furthermore, advances in intraoperative imaging and broader adoption of standardized AR techniques may enhance surgical precision and extend the oncologic benefit of AR. Ultimately, reducing NTR may not only improve long-term survival, but also preserve eligibility for salvage therapies, thus reinforcing the critical role of surgical strategy in the overall management of HCC.
